# Effects of two nAChR agonists on wood ants: acetamiprid induces lethality and immediate hypoactivity, while flupyradifurone causes time-delayed hyperactivity

**DOI:** 10.1007/s11356-025-36755-z

**Published:** 2025-07-18

**Authors:** Leonie Scheibli, Sabrina Pia Geiger, Sarah Elisabeth Pfeffer

**Affiliations:** Institute of Neurobiology, Ulm University, 89081 Ulm, Germany

**Keywords:** Bioassay, Butenolide, Neonicotinoids, Insecticide, Behavior, Insect decline, Hymenopterans

## Abstract

Neonicotinoids and related compounds, such as acetamiprid and flupyradifurone, are widely used in agriculture to control pest insects. Due to their broad mode of action, their effects are not restricted to target species, potentially causing harm to other animals as well. Investigating these effects on non-target organisms is crucial for environmental protection, biodiversity conservation, and ecosystem sustainability. In the present study, we examined lethal and sublethal effects of acetamiprid and flupyradifurone on wood ants (*Formica polyctena*), a key species in wood ecosystems, within a dose range of 0–30 ng active substance/mg body weight (ng a.s./mg bw). Acetamiprid significantly reduced survival at doses above 143 ng/individual (equivalent to > 10 ng a.s./mg bw) with an LD50 of 323.4 ng/individual at 72 h (equivalent to 23.1 ng a.s./mg bw). Moreover, acetamiprid induced tremors and comatose states and restricted ant mobility with significantly reduced distances and lower walking speeds. Flupyradifurone exhibited less severe effects on survival and physical condition but induced a time-delayed hyperactivity 48 to 72 h post-treatment. In summary, these findings suggest distinct impacts of two nAChR agonists, acetamiprid and flupyradifurone, on *Formica* wood ants and emphasize the importance for species-specific research across a diverse array of organisms, particularly in the context of risk assessment.

**Supplementary Information:**

The online version contains supplementary material available at 10.1007/s11356-025-36755-z.

## Introduction

Neonicotinoids and neonicotinoid-like compounds, such as acetamiprid and the more recently introduced butenolide flupyradifurone, are widely used pesticides due to their effectiveness against agricultural pests such as aphids, plant- and leafhoppers, bugs, and whiteflies (Jeschke et al. 2011, 2015; Klingelhöfer et al. 2022). However, these substances have raised significant concerns due to their non-selective mode of action and impact on non-target insects, particularly beneficial insects like hymenopterans (Desneux et al. 2007; Bonmatin et al. 2015; Pisa et al. 2015; Singla et al. 2021; Mamy et al. 2023). These chemicals act as agonists of nicotinic acetylcholine receptors (nAChRs), meaning they bind to and activate these receptors, mimicking the action of the natural neurotransmitter acetylcholine. nAChRs play crucial roles in the insect nervous system, and the disruption of neuronal transmission by these agonists can lead to paralysis and death at high doses, while low doses can induce various sublethal effects, for example, altered mobility, memory, or learning performances (Desneux et al. 2007; Tomizawa 2013; Casida 2018; Pereira et al. 2020). Despite targeting the same site in insects, acetamiprid and flupyradifurone exhibit distinct chemical properties due to differences in their key pharmacophore structures. Acetamiprid contains an N-cyano-amidine group, while flupyradifurone features a butenolide group. These structural differences, along with their specific substituents, significantly influence their interactions with nAChRs in the insect nervous system (Jeschke et al. 2015). These structural differences determine their binding affinities, selectivity, and ultimately their neurotoxic effects, leading to distinct behavioral and physiological outcomes in target species (Jeschke et al. 2015; Casida 2018). Moreover, these chemical differences play a critical role in how acetamiprid and flupyradifurone are metabolized and detoxified in insects. Each pesticide is processed through distinct enzymatic pathways, with detoxification systems like cytochrome P450 enzymes playing a central role. However, predicting detoxification rates across species is challenging due to the complexity of P450 biochemistry and the extensive diversity of these enzymes in arthropods (Jeschke et al. 2015; Nauen et al. 2022). These factors underscore the importance of understanding species-specific biochemical and physiological responses to these pesticides.

Doubts regarding the environmental safety of neonicotinoids have prompted regulatory measures within the European Union (EU), particularly targeting substances associated with elevated risks to crucial pollinator populations, such as honeybees or bumble bees (EU 2018)/783; EU 2018)/784; EU 2018)/785). Acetamiprid and flupyradifurone are excluded from these restrictions, mainly due to (supposed) lower ecotoxicological risk profiles compared to other neonicotinoids (Nauen et al. 2015; Feyereisen 2018). Both substances have been shown to exert less severe effects on honeybees than other neonicotinoids, such as imidacloprid, clothianidin, or thiamethoxam (Nauen et al. 2015; Pisa et al. 2015). However, most research focuses on honeybees, leading to a significant bias in available data (Dirilgen et al. 2023). Extrapolating pesticide risks from honeybee studies to other insects is problematic (Siviter and Muth 2020)—even within the order Hymenoptera, since the effects of pesticides can vary strongly among species (Siviter et al. 2021). The lack of data transferability underscores the need for species-specific research. Although ants belong to the Hymenoptera order, the potential impacts of pesticides, including nAChR agonists, on these insects remain remarkably underexplored (Sorvari 2016). This knowledge gap is particularly pronounced for ecologically significant native species like wood ants, as research efforts have predominantly focused on controlling invasive ant species (Schläppi et al. 2021). Previous studies on sublethal effects of neonicotinoids in ants have revealed adverse impacts on various behaviors and physiological processes, including activity and locomotion, grooming, competitive interactions, nest-building, food consumption, foraging behavior, reproduction, and colony growth (Galvanho et al. 2013; Barbieri et al. 2013; Thiel and Köhler et al. 2016; Sappington 2018; Schläppi et al. 2020; Frankel and Frankel 2021; Frizzi et al. 2023, Svoboda et al. 2023).

Wood ants like *Formica polyctena* (Hymenoptera, Formicidae; Förster, 1850) are keystone species and ecosystem engineers in many forest environments, contributing to their health and stability through several ecological roles (Sorvari 2016). They often build large interconnected colonies with multiple nest sites, composed of subterranean chambers and above-ground mounds of pine needles, small branches, and other organic matter collected from the surroundings (Ellis and Robinson 2014). Wood ants aid in soil aeration and fertilization, pest control, seed dispersal, and are integral parts of the food web (Berberich et al. 2020; Stockan and Robinson 2016). Their presence and abundance serve as indicators of environmental health, with robust wood ant populations often reflecting well-balanced and biodiverse ecosystems (Ellison 2012; Chen and Robisnson 2014; Berberich et al. 2017; Skaldina et al. 2018). Despite their ecological importance in temperate and boreal forests of Eurasia, wood ants face numerous threats, and there is increasing evidence of local declines and extinctions (Balzani et al. 2022). Dekoninck et al. (2010) reported a decline of more than 50% of red wood ant colonies in the northwestern part of Belgium over a time course of 20 years. A similar trend was described for the Netherlands, where a significant reduction of *F. polyctena* nests was observed from 1986 to 2014 (Mabelis 2016). These declines are attributed to several factors, like habitat loss or urbanization (Sorvari 2016). However, intensive agriculture and extensive pesticide use are most likely key drivers behind the loss of *Formica* colonies, though this aspect remains underexplored in current research (Mabelis 2016; Sorvari 2016; Pohl et al. 2024).

Toxicological studies predominantly focus on honeybees, but their findings cannot be directly transferred to other arthropods, including Hymenoptera due to interspecies differences in physiology or behavior (Dirilgen et al. 2023; Siviter et al. 2021). Notably, wood ants have rarely been addressed regarding potential effects of pesticides, despite their ecological importance (Sorvari 2016). Furthermore, risk assessment studies predominantly focus on contact intoxication (EFSA PPR 2015; Pohl et al. 2024), often overlooking oral exposure pathways. This is particularly significant for ants, where food sharing through trophallaxis is a critical mechanism for resource distribution and colony communication (Josens et al. 2016). Therefore, this study aims, for the first time, to investigate the lethal and sublethal effects of two acute orally applied nAChR agonists, acetamiprid and flupyradifurone, on *F. polyctena*. Ants were treated with a single dose of 0–30 ng active substance/mg body weight (ng a.s./mg bw) (corresponding to 20–400 ng/individual) and monitored over 336 h. Our bioassay analysis focused on assessing basic vitality parameters, specifically physical condition and mobility. This research aims to provide critical insights into the impact of neonicotinoids and neonicotinoid-like pesticides on wood ants and provide reproducible bioassay data relevant for ecotoxicology, potentially contributing to a broader understanding of their ecological implications.

## Material and methods

### Animal care

Wood ants (*Formica polyctena*) were obtained from a large nest in their natural habitat near Tomerdingen (48°29′52.8″ N 9°52′43.4″ E) in 2021. The ants were acclimatized in the laboratory for 3 days and kept under controlled conditions with constant temperature (23 ± 2 °C), humidity (70–80%), and photoperiod (16:8 light:dark, simulating summer time condition). The ants were housed in plastic boxes with air holes and spruce needles to mimic their natural habitat. Experiments that required individual monitoring (survival probability and physical condition) were conducted with ants kept separately, while for experiments involving mobility, ants were housed in groups of five. Although housing in groups of more than 10 individuals is preferable for ant maintenance (Koto et al. 2023), we chose individual or small group housing due to experimental constraints, including disruptions of nestmate recognition from individual marking and the need to minimize identity swaps during EthoVision tracking. Water and honey water were provided ad libitum throughout the entire experimental procedures. Prior to each trial, ants exhibiting signs of poor health, including abnormal behavior, limb deficiency, or unresponsiveness, were excluded from the experiments. All experiments were conducted in compliance with the current German laws and ethical guidelines of Ulm University.

### Pesticide solution and application

In our investigation, ants were fed with specific doses of pesticide-contaminated sucrose solution to accurately determine the amount of ingested acetamiprid (Sigma-Aldrich, PESTANAL, purity: ≥ 98.0%) and flupyradifurone (Sigma-Aldrich, PESTANAL, purity: ≥ 98.0%). Specific target doses (20 ng, 40 ng, 60 ng, 80 ng, 100 ng, 150 ng, 200 ng, 300 ng, 400 ng) were diluted in 0.5 µL of 50% (w/v) sucrose solution. A pure 50% (w/v) sucrose solution served as the control. The target doses (in ng) were selected based on preliminary experiments and the variability in the ants’ body weight (for details, see below). While these doses are relatively low and likely within the range that wood ants may encounter in their natural environment, there is currently no available data on specific oral contamination loads in *Formica* ants (see also section “Contamination environment of wood ants” in the discussion section).

Prior to the experiment, each individual ant (of every experimental control and test group) was weighed (Sartorius, MC210P) to normalize the administered pesticide dose to its body weight in order to minimize the impact of size variations. The mean body weight across different control and treatment groups ranged between 11.91 and 15.04 mg (grand mean: 13.7 mg). Due to this normalization, pesticide-treated ants were assigned to one of the following dose ranges: > 0–10 ng a.s./mg bw, > 10–20 ng a.s./mg bw, and > 20–30 ng a.s./mg bw. For more detailed information on group assignment, see Supplementary Table S1. To enhance intra- and inter-specific comparability, we express dose levels relative to the ants’ body weight (ng a.s./mg bw); however, since insect toxicity is historically often reported in individual units (ng/individual) (Thompson and Pamminger 2019), we have included both expressions in our discussion part.

For the oral pesticide treatment, each ant was individually fed a single 0.5 μL droplet of the pesticide or control solution. Ants were gently held by their hind legs, and a pipette (Eppendorf, ResearchPlus 0.1–2.5 μL) was used to apply the respective control or test solution directly between their mandibles. We waited until the ant consumed the entire sugary droplet. After ingestion, the ants were returned to their housing boxes. Only individuals that fully consumed the droplet were included in the experiment. This feeding method ensured precise control over the amount of pesticide ingested by each ant.

### Survival probability and LD50 calculation

The survival of *Formica* wood ants was assessed after oral treatment with a specific dose of acetamiprid or flupyradifurone, ranging from 0 to 30 ng a.s./mg bw (see application procedure above). An ant was declared dead if no movement was observed upon contact stimulation (gentle touching with a fine brush). Survival was recorded immediately after exposure (0 h; 2 h) and subsequently at 24-h intervals up to 336 h (14 days) post-treatment. We do not present data beyond that time frame because (i) as expected, only minimal changes in survival were observed after such a relatively long period following a single oral treatment, and (ii) survival in the control group declined below 80% and drawing conclusions regarding pesticide effects would be ambiguous.

### Physical condition

**Fig. 1:**
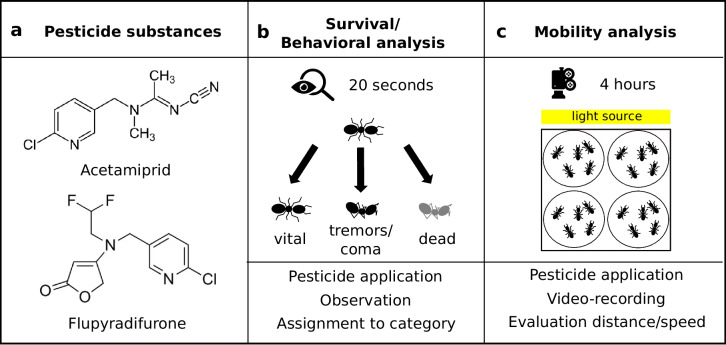
Overview of the experimental setup. **a** Insecticides used in the present study. Schematic procedure representation for **b** survival and behavioral analysis and **c** mobility analysis

To assess the specific physical condition of each ant, observations were conducted for a 20-s period at each time point (Fig. 1), namely, immediately following pesticide exposure (0 h; 2 h) and subsequently at 24-h intervals up to 336 h post-treatment. Various states were assigned to describe the condition of each insect: “vital,” “tremor with righting response,” “tremor without righting response,” or “coma.” Ants classified as *vital* displayed no abnormal behavior, responded to contact stimuli, and demonstrated normal walking capabilities. An ant was categorized as *tremor with righting response* if it could move despite trembling movements of the antennae or extremities. Although frequent falling was often observed in this category, the ant retained the ability to right itself. Ants exhibiting trembling movements but unable to move further or, in severe cases, lying on their backs were categorized as *tremor without righting response*. Finally, *comatose* ants showed a lack of spontaneous movement, yet upon contact stimulation, subtle movements of legs, antennae, or mouthparts were observed. Following the determination of each ant’s current state, they were returned to their respective housing boxes and accommodated as described in section “Animal care.” Dead ants were further observed to validate their group assignment, as comatose ants occasionally displayed signs of recovery and subsequently regained mobility, even if initially declared deceased.

### Mobility analysis

To evaluate the mobility of ants, their movements were recorded over a 4-h period at four distinct time points (1, 24, 48, and 72 h) following pesticide treatment (see Fig. 1). Each test group comprised five ants and was placed in a filming arena with a diameter of 8.5 cm. Prior to recording, the ants were allowed to acclimate within the arena for 10 min. The setup was positioned under a Basler acA1300-60gm camera, with dimmed light sources ensuring optimal lighting conditions. Between recording intervals, all ants were returned to their housing boxes following the procedures outlined in section “Animal care.” Video tracking and mobility analysis were conducted using EthoVision XT15 software from Noldus, Wageningen, Netherlands. The position of each ant was tracked six times per second throughout the entire 4-h recording period. The trajectories of ants were analyzed in terms of total distance traveled (m) and time course of walking speed (cm/min). To minimize identity switches during tracking, we (i) optimized EthoVision’s tracking parameters by applying the software’s filter function to identify and exclude improbable movements based on distance thresholds per unit time and (ii) used groups of five ants for tracking to provide reliable mobility assessments.

### Statistics

Statistical analyses were carried out with RStudio Version 4.0.5 (R Core Team 2024), and the R code used is included in the Supplementary Material.

For *survival probabilities*, illustration was realized by Kaplan–Meier curves in R (survival package: Therneau 2000). A logit model (ecotox package; Wheeler et al. 2006) and statistical comparisons were performed by a log-rank test for pairwise comparison with Bonferroni adjusted *p*-values (survival package: Therneau 2000). The coxph function of the survival package (Therneau 2000) was used to calculate the hazard ratios and the Concordance Index. A logit model (ecotox package; Wheeler et al. 2006) was used to calculate LD50 values at 72 h. The time point was chosen when the mortality rate dropped below 50% in acetamiprid treatment, which is a favorable criterion for LD50 calculation (see OECD No. 213, 1998 or OECD No. 247, 2017). We used the dose in ng a.s./mg bw to calculate the respective LD50 and subsequently transformed the value in dose/individual by using the grand mean of all body weights (14.0 mg).

For *physical condition*, the statistical significance of pesticide treatment on the frequency of abnormal behaviors (defined as sum of tremors (with and without righting response) as well as comatose states) was accomplished by using a generalized linear mixed model (GLMM). Time and treatment without interaction were determined as fixed effects, subject ID as random factor, and we used a binomial distribution with a logit link function for the analysis (lme4 package: Bates et al. 2015). To improve interpretability as well as prediction accuracy, a lasso regression was used; *p*-values were assessed using bootstrapping. As flupyradifurone induced only a few abnormal behaviors, it was not possible to fit a GLMM; therefore, a generalized linear model (GLM) was applied. Here, time and treatment without an interaction term were used for model specification.

For *total distance covered*, statistics were realized using a linear mixed-effect model (LMM; lme4 package: Bates et al. 2015) with treatment and time as fixed effects with an interaction term and the ant IDs as random factors. As the assumptions for a linear mixed effect model were not met for all cases, we used a robust alternative (*robustlmm* package: Koller 2016). Here, the AIC and BIC as well as ANOVAs were used to compare model fits. We used the Cohen’s *d* to report effect sizes for those mobility models. The effect sizes were classified according to the following criteria from trivial to strong effect: Cohen’s *d *0–0.2: trivial; > 0.2–0.5 small, > 0.5–0.8 moderate, and > 0.8 strong effect. We used those keywords in the description of the results (Blanar et al. 2009).

We used a total of 315 wood ants in our experiments, with 155 assigned to the investigation of survival and physical condition, and 160 to the mobility analysis. Each test or control group was initially assigned 20 ants; however, due to variations in feeding success (see section “Pesticide solution and application”) and the inclusion of only fully exposed ants, the final group size varied slightly (18–21 ants). To minimize invasive interventions for species protection and due to the time-intensive nature of individual pesticide application, only a limited number of wood ants were collected, all from a single nest site. This approach, while necessary, may introduce potential bias into the data. Despite the small sample size, we are still confident that results provide robust insights into survival trends and reflect meaningful patterns of sublethal effects.

## Results

### Survival probabilities

**Fig. 2:**
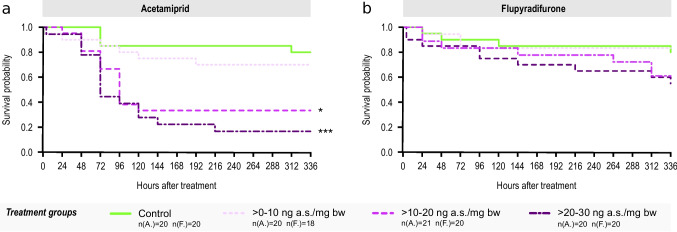
Survival analysis for *Formica* wood ants (*F. polyctena*) after oral treatment with single doses of acetamiprid (**a**) or flupyradifurone (**b**). Survival probabilities are illustrated with Kaplan–Meier survival curves; different dose ranges are shown in different colors and line types. Pairwise comparison to control was realized via log-rank test and Bonferroni adjusted *p*-values. The legend includes sample size information, with A. representing acetamiprid and F. representing flupyradifurone. For raw data, see supplementary Table S2

Oral ingestion of acetamiprid or flupyradifurone (both 0–30 ng a.s./mg bw in three dose ranges) had different effects on the survival of *Formica* wood ants (Fig. 2). While there were statistically significant differences from the control in the two highest acetamiprid doses of 10–20 ng a.s./mg bw (log-rank test, *p < 0.05) and 20–30 ng a.s./mg bw (log-rank test, ***p < 0.001), the lowest treatment group showed a survival probability comparable to the control (log-rank test, *p* = 1.0). Ants exposed to the 10–20 ng a.s./mg bw treatment group were five times more likely to die, whereas those in the 20–30 ng a.s./mg bw group exhibited an even sevenfold increase in the risk of death. The Concordance Index (*C* = 0.71) indicates a moderate effect size for the predictive ability of the model. The calculated LD50 value at 72 h for acetamiprid was determined at 23.1 ng a.s./mg bw (LCL: 14.4 ng a.s./mg; UCL: 218 ng a.s./mg), corresponding to 323.4 ng/individual. For flupyradifurone treatments, there were no statistically significant differences to control; however, the two highest treatment groups exhibited a slightly higher mortality risk compared to the control (> 10–20 ng a.s./mg bw: 2.6 times higher hazard; > 20–30 ng a.s./mg bw: 2.1 times higher hazard) (log-rank test, *p* > 0.6; in all test groups, Concordance Index: 0.63), and the recommended 50% mortality rate was not exceeded (e.g., OECD 2017); therefore, no LD50 was determined. Control mortality falls within the acceptable range for testing insect survival as specified by OECD guidelines (e.g., OECD 1998).

### Physical condition

**Fig. 3:**
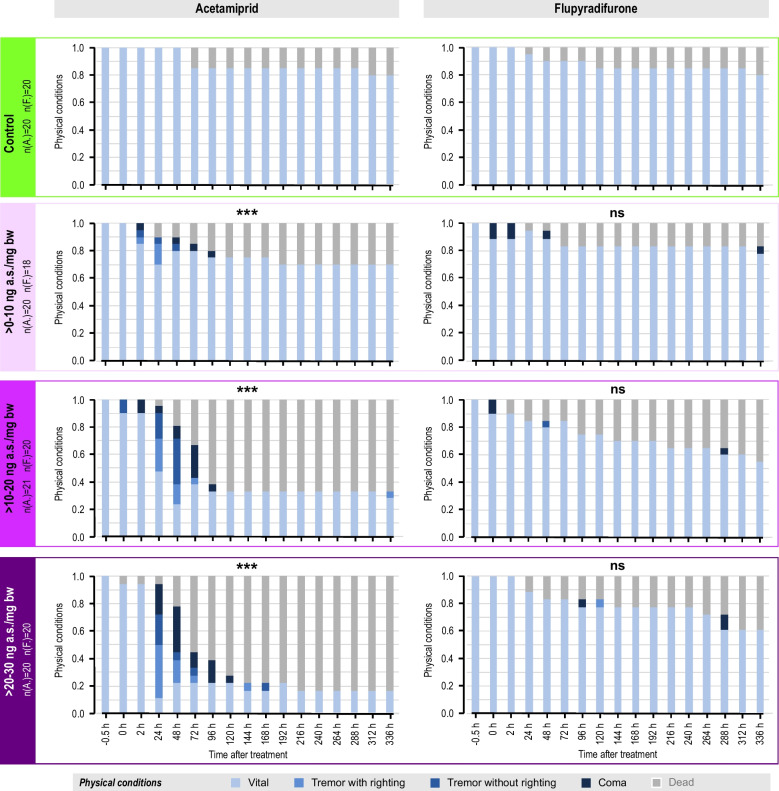
Effects on physical condition of *Formica* wood ants (*F. polyctena*) after single oral treatments with acetamiprid (left) or flupyradifurone (right). Increasing pesticide dose levels from top to bottom. Note that pesticide application was directly before time point “0 h”; the time point before treatment (− 0.5 h) served as an additional control. At every observation time point, each ant was assigned to one of the following physical condition states: *vital, tremor with righting response, tremor without righting response*, or *coma*. Additionally, dead individuals are added in the graph to depict all ants of each test group. Statistical analysis was realized via GLMM (acetamiprid) or GLM (flupyradifurone); significant differences to control are highlighted by asterisks in respective pesticide-treated group (ns, not significant; ***p < 0.001). The heading on the left side includes sample size information, with A. representing acetamiprid and F. representing flupyradifurone. For raw data, see supplementary Table S3

In the control group, all ants alive remained in a “vital” state throughout the entire observation period (Fig. 3). Both acetamiprid and flupyradifurone caused impairment of the physical condition of *Formica* wood ants. Especially, acetamiprid treatment revealed a significant dose-dependent effect, with higher doses showing a time-delayed increase of abnormal states like tremors and coma (GLMM, ***p < 0.001; in all test groups). A large percentage of acetamiprid-treated ants died towards the end of the observation period (30%, 67%, 83% survival rate of test groups for > 0–10 ng ng a.s./mg, > 10–20 ng a.s./mg bw, > 20–30 ng a.s./mg, respectively). Compared to acetamiprid, flupyradifurone had less serious consequences regarding the ants’ physical conditions, with sparsely emerging abnormal states and a slight tendency of an increased mortality during the experimental period (see also Fig. 2). Flupyradifurone did not significantly increase the frequency of abnormal behaviors in *Formica* wood ants (GLM, *p* > 0.9; in all test groups).

### Mobility

**Fig. 4:**
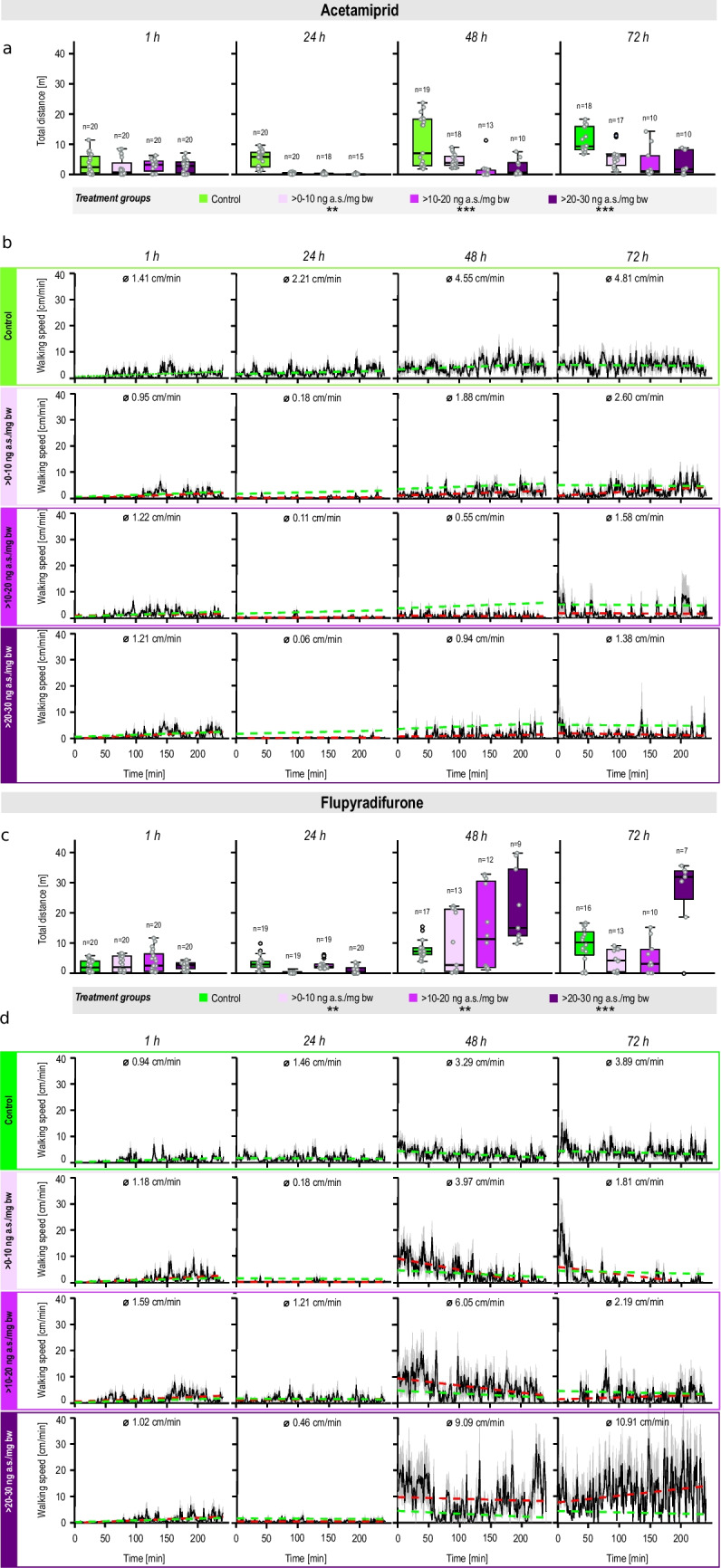
Effects on total distance covered (**a**, **c**) and on time course of walking speed (**b**, **d**) after single oral treatments with acetamiprid (above) or flupyradifurone (below). Total distance covered during 4 h recording at specific time points (1 h, 24 h, 48 h, 72 h) after pesticide treatment (**a**, **c**). Statistical analysis of covered distances using a LMM with significant differences to control (*p < 0.05; **p < 0.01; ***p < 0.001). Note that sample size information for mobility analysis is provided in **a** and **c** above each box plot. The course of walking speeds (**b**, **d**) during 4-h recording is given in centimeters per minute. Numbers in each subplot illustrate the mean walking speed recorded over a 4-h observation period. The black line shows the mean covered distance per ant, and the gray shadows depict the standard errors of the means. The green line depicts the trend of the velocity over time for the control, whereas the red line depicts this trend for the treatment group at the respective time point. For better comparison, both lines are shown in the treatment groups. For raw data, see supplementary Table S4

For control ants, total distance covered and walking speed profiles remained relatively stable and consistent over the entire recording period at all observed time points (Fig. 4). We interpret the slight increase in distance walked over time in the control group as reflecting natural behavioral adjustments to setup and handling procedure. Contrarily, acetamiprid treatment resulted in reduced mobility in a dose-dependent manner, with shorter total distance covered and slower walking speeds (LMM; **p < 0.01 for > 0–10 ng a.s./mg bw; ***p < 0.001 for > 10–20 ng a.s./mg bw and > 20–30 ng a.s./mg bw). The effect was most pronounced after 24 h, with the control group covering a median distance of 5.82 m, whereas the pesticide-treated ants only covered distances ranging between 0.09 and 0.41 m (> 0–10 ng a.s./mg bw: 0.41 m; > 10–20 ng a.s./mg bw: 0.25 m; > 20–30 ng a.s./mg bw: 0.09 m). Acetamiprid-treated ants exhibited slight signs of recovery after 72 h; however, their mobility levels did not fully return to those observed in the control group at the corresponding time point. These findings were supported by the representation of the mean running speeds. It was observed that the control group exhibited an increasing trend from 1.41 cm/min (1 h) to 4.81 cm/min (72 h), whereas the acetamiprid-treated ants achieved lower mean speeds. The lowest speeds in the pesticide-treated group occurred 24 h after treatment (> 0–10 ng a.s./mg bw: 0.18 cm/min; > 10–20 ng a.s./mg bw: 0.11 cm/min; > 20–30 ng a.s./mg bw: 0.06 cm/min). Regarding distances covered, effect size (Cohen’s *d*) of the lowest acetamiprid treatment was moderately negative (− 0.70), whereas the two higher treatment groups showed only small negative effect sizes (> 10–20 ng a.s./mg bw: − 0.30; > 20–30 ng a.s./mg bw: − 0.20). The effect sizes for interaction between time and treatment were small (− 0.02 for the lowest and − 0.04 for the two higher treatment groups) for all acetamiprid treatment groups.

Flupyradifurone treatment induced statistically significant changes in mobility metrics compared to controls, with a time-delayed (48 h, 72 h) hyperactivity, especially evident at the highest dose (LMM; **p < 0.01 for > 0–10 ng a.s./mg bw and > 10–20 ng a.s./mg bw; ***p < 0.001 for > 20–30 ng a.s./mg bw). Here, the control group covered a median distance of 7.32 m (48 h) and 10.27 m (72 h), whereas flupyradifurone-treated ants at doses with > 20–30 ng/mg showed longer distances with 14.97 m (48 h) and 31.76 m (72 h). This hyperactive behavior was also evident in mean speeds, with the highest values recorded in the highest flupyradifurone treatment group with 9.09 cm/min (48 h) and 10.91 cm/min (72 h). In contrast, the control ants reached a maximum speed of 3.69 cm/min (72 h). Effect sizes for flupyradifurone were trivial or small, and therefore, the effects were less strong than in acetamiprid (> 0–10 ng a.s./mg bw: 0.00; > 10–20 ng a.s./mg bw: 0.20; > 20–30 ng a.s./mg bw: − 0.30). These findings suggest that while both pesticides impact ant mobility, their effects manifest differently, with acetamiprid causing more immediate and pronounced reductions in activity, and flupyradifurone potentially inducing delayed hyperactive responses.

## Discussion

### Contamination environment of wood ants

Pesticide studies regarding lethal or sublethal impacts on wood ants are exceptionally rare (Sorvari et al. 2016), and the relative importance of contamination routes and corresponding potential pesticide loads still remain to be investigated (Schläppi et al. 2021; Pohl et al. 2024). However, the specific lifestyle of *Formica* wood ants makes them prone to long-term contamination through pesticides. Due to a polydomous (“many nest sites”) and polygynous (“many queens”) colony mode of *F. polyctena,* they are a territorial ant species with remarkable longevity (Czechowski and Vepsäläinen 2009). Thus, they may face a chronic contamination within their nesting area and a potential acute exposure during foraging or nest-building activities (Schläppi et al. 2021). Wood ant colonies are frequently established along forest edges, where agricultural areas may be close and ants are exposed to pesticides with increased probability (Zmihorski 2010; Sorvari et al. 2016). Unintentional exposure routes of neonicotinoids and similar substances include contact with spray droplets, contaminated soil and water, or potential nest-building material (needles, small twigs, resin, etc.) (Sorvari 2013; Bonmatin et al. 2015; Schläppi et al. 2021).

As wood ants are central-place foragers and have an omnivorous diet (Domisch et al. 2016), oral ingestion of pesticide substances is possible via contaminated arthropod prey or polluted plant material such as nectar, pollen, seeds, guttation fluid, or honeydew secreted by aphids (Domisch et al. 2016). Although ants have developed remarkable mechanisms to avoid specific baits (Zanola et al. 2024), the widespread presence of agriculturally applied pesticides makes complete avoidance nearly impossible once agrochemicals are introduced into the environment. Notably, neonicotinoids have been shown to potentially increase the intake of contaminated food, as observed in bees (Kessler et al. 2015). Oral ingestion of pesticide-treated prey items, such as dead or weakened arthropods, readily available in large amounts near intensively used agricultural landscapes, represents a significant exposure route for ants (Pohl et al. 2024). Moreover, efficient nutritional transmission through trophallaxis may further distribute pesticide-contaminated material at the colony level (Jung et al. 2018). Case reports have documented instances where specific wood ant colonies near agricultural fields have disappeared (Sorvari et al. 2016). On a larger scale, declines in *Formica* wood ant colonies, well documented in western Europe, have identified intensive agriculture and extensive pesticide use as key drivers (Dekoninck et al. 2010; Mabelis 2016).

### Acetamiprid reduced survival of *Formica* wood ants

Wood ants showed a higher sensitivity to oral ingestion of acetamiprid compared to flupyradifurone. In our experiments, an oral LD50 value for acetamiprid was determined at 323.4 ng/individual (equivalent to 23.1 ng a.s./mg bw) at 72 h post-treatment, while for flupyradifurone, the examined dose range, between 20 and 400 ng/individual (equivalent to 0–30 ng a.s./mg bw), did not significantly alter *F. polyctena* survival.

A similar tendency was detected for bumble bees that have been shown to be more susceptible to acetamiprid than to flupyradifurone. Oral LD50 values for acetamiprid were reported to range between 2.1 and 2.8 ng/bumble bee (0.0021–0.0028 µg a.s./bumble bee) (Wu et al. 2010), while for flupyradifurone, a value of 2823 ng/bumble bee (2.823 µg) was estimated (Mundy-Heisz et al. 2022). Interestingly, a reversed pattern of sensitivities was observed in honeybees with an oral LD50 value of 8850 ng/honeybee (8.85 µg/honeybee) (Capela et al. 2022) for acetamiprid and a value between 1200 ng/honeybee (1.2 µg, Nauen et al. 2015) and 3368 ng/honeybee (3.368 µg, Gao et al. 2023) for flupyradifurone. Notably, both LD50 values for honeybees can be rated as relatively high by comparison. These findings underscore the variability in pesticide sensitivity among different hymenopteran species and highlight the need for species-specific toxicological assessments. Data on honeybees (or any single model species) cannot sufficiently provide a general estimate of insect vulnerability to pesticides.

While our findings suggest that flupyradifurone has limited harmful effects on wood ants within the tested dose range, caution is still warranted in drawing definitive conclusions about its overall safety. We specifically examined a lower dose range, and the potential effects of higher doses, which could occur in natural settings, remain unclear. This is particularly important given flupyradifurone’s classification as “bee-friendly,” which permits application schemes and time points that differ from other pesticides, including its use on flowering crops (Tosi et al. 2021). Our study was based on a single oral dose under controlled laboratory conditions with ad *libitum* food and water. However, in field conditions, chronic exposure through multiple contamination pathways is more likely, making it difficult to predict the potential colony-level effects.

### Acetamiprid induced immediate tremor, comatose states, and hypoactivity, while flupyradifurone caused time-delayed hyperactivity

After oral exposure to acetamiprid or flupyradifurone, *Formica* wood ants displayed distinct behavioral and physiological responses, highlighting the prominent but contrasting effects of these pesticides. Acetamiprid caused immediate and pronounced physical deterioration, characterized by tremors, comatose states, and significantly reduced mobility within 24 h post-treatment and a slight recovery effect after 72 h. In contrast, flupyradifurone elicited a delayed response, with increased mobility observed between 48 and 72 h after application. Although walking in a round arena represents an artificial behavior that does not fully replicate natural foraging or field conditions, the observed effects are significant and reproducible in a time-and-concentration-dependent manner. These mobility changes are consistent with previously documented sublethal effects of neonicotinoids and related compounds (see next paragraph) and offer valuable insights into their neurotoxic impacts. Impaired walking, a crucial behavior for activities like foraging, escaping, or exploring, could potentially affect colony-level efficiency and survival (Gill et al. 2012; Dürr et al. 2018). However, these potential effects remain speculative, and we cannot draw definitive conclusions regarding their relevance in natural settings.

Constraints of motor function, such as uncoordinated movements, tremors, hyperactivity, hypoactivity, and even knockdown, have frequently been reported as sublethal side effects of nAChR agonists (Desneux et al. 2007). For honeybees and other hymenopterans, pesticides like imidacloprid, thiamethoxam, acetamiprid, or flupyradifurone have been shown to increase the frequency of abnormal motor behavior (Suchail et al. 2001; Williamson et al. 2014; Tosi and Nieh 2017; Hesselbach and Scheiner 2019; Jacob et al. 2019; Ludicke and Nieh 2020). Similar motor impairments caused by nAChR agonistic pesticides have also been detected in non-hymenopteran species like beetles, lacewings, mayflies, or cockroaches (Wen and Scott 1997; Bartlett et al. 2018; Scheibli et al. 2023, 2024), and even in “higher-order animals” including mammals (Anadón et al. 2020; Dardiotis et al. 2023). Motor function constraints (see also below) are suitable parameters for assessing sublethal effects of pesticides like neonicotinoids and similar substances on non-target insects, as they are easily accessible, more sensitive than lethality, and detectable across a wide range of different species. Analyzing behavioral endpoints related to motor function and mobility can thus yield valuable insights into the ecological implications of these pesticides on non-target insect species.

### Molecular differences and its implication on specific effects of acetamiprid and flupyradifurone

Both pesticides target nAChRs in the ant’s nervous system and were applied with the same doses; however, induced effects differed notably between the two substances in *Formica* wood ants. Neonicotinoids and other nAChR agonists can indeed have both hyperactive (stimulant) and hypoactive (sedative) effects in insects, depending on the chemical substance, its applied dose, or the time frame after treatment (Blacquière et al. 2012; Simon-Delso et al. 2015). Additionally, insecticide-specific detoxification mechanisms, such as those mediated by cytochrome P450 enzymes, are likely key factors influencing different susceptibilities. However, to the best of our knowledge, these pathways have not been investigated in wood ants and may vary across species. Nevertheless, studies in bees have demonstrated that there is a 50% clearance of flupyradifurone within 24 h of contact application (Haas et al. 2021). This suggests that the time-delayed effects observed in the present study could be attributed to the persistence of flupyradifurone within the ants’ system, remaining active between 24 and 72 h post-exposure. Specific behavioral effects may be induced directly by flupyradifurone or by its metabolic products, although the latter have been reported as non-toxic to bees (Haas et al. 2021).

Acetamiprid and flupyradifurone differ in their chemical properties and modes of action. Acetamiprid contains an N-cyano-amidine pharmacophore system, while flupyradifurone is characterized by a butenolide pharmacophore system (Jeschke et al. 2015; Casida 2018). Unique substituents in the two pesticides further refine their molecular interactions and toxicological profiles, and in particular, flupyradifurone exhibits an additional interaction with a specific tyrosine residue in the nAChR, involving hydrogen-bond-like connections, which helps explain its differences from other neonicotinoids (Beck et al. 2015). The chemical differences contribute to the specific physicochemical properties, insecticidal activity, or metabolism in insects (Jeschke et al. 2015). However, the exact binding site affinities are not fully understood and are difficult to predict for particular species like *F. polyctena*. The target nAChRs can differ strongly between insect species due to the presence of multiple combinations of different nAChR-subunits, while alternative splicing and RNA editing further enhance the diversity of receptor structures (Ihara et al. 2017; Lu et al. 2022). Furthermore, various pesticides undergo different biotransformations to toxic metabolites, which can lead to prolonged pesticide effects (Suchail et al. 2001; Simon-Delso et al. 2015). This mechanism could also explain the time-delayed hyperactivity in flupyradifurone-treated ants. Altogether, these findings suggest that, although both pesticides impact nAChRs in *Formica* wood ants, their effects diverge and manifest differently, with acetamiprid causing a more immediate reduction of mobility and flupyradifurone inducing a time-delayed hyperactivity within the applied dose range. Predicting such outcomes through theoretical approaches beforehand remains challenging due to the presence of unknown species-specific molecular mechanisms, further emphasizing the importance of investigations on diverse non-target organisms.

### Conclusion

Acetamiprid and flupyradifurone - while both targeting the same receptor types (nAChRs)—exhibit significantly different effects on *Formica* wood ants. Acetamiprid caused immediate and severe impairments, including decreased survival, compromised physical condition, and reduced mobility. In contrast, flupyradifurone had less severe effects but elicited time-delayed hyperactive behavior.

Though similar effects have been observed with other nAChR agonists across various insect species, the effective dose ranges vary significantly. There is a challenge of extrapolating risk assessment data from few species to other non-target organisms, which may not be sufficient to provide general estimates of insect vulnerability to particular pesticide substances. Currently, there are few surrogate species (e.g., honeybees) and safety factors (normally ranging between 2 and 100) to address uncertainties in pesticide risk assessment (Milner and Boyd 2017; Topping et al. 2020). It is crucial to address such uncertainties as our experiments highlight the differences in pesticide susceptibility even within a single taxon, such as hymenopterans.

Effective protection of arthropods requires expanding research to include a broader range of species and collecting more data on non-target organisms. Given the impracticality of studying every insect species, selecting representative species based on their ecological roles and pesticide susceptibility is essential (Topping et al. 2020). It has already been proposed to include ants as key representatives of soil-dwelling arthropods in future risk assessment procedures (Schläppi et al. 2021).

Despite the differences in the effective dose level just mentioned, sublethal effects regarding motor function and mobility patterns are surprisingly similar across different pesticides and species. However, current risk assessments primarily focus on lethal effects (Tosi et al. 2022). Even though EU Regulation (EU No 1107/2009) states that an active substance should not pose an intolerable threat to honeybee behavior—and in general to non-target organisms—there are still no standardized methods to examine behavioral effects (Tosi et al. 2022). Analyzing behavioral endpoints, such as impaired motor function and mobility, could provide valuable insights for the framework of risk assessment and address broader ecological implications of these pesticides on non-target insect species.

Finally, risk assessment studies predominantly emphasize contact toxicity (EFSA PPR, 2015; Jansen 2012), yet real-world exposure is far more complex. Insects can also encounter pesticides through oral ingestion and inhalation, both of which can have profound impacts on their health (Pohl et al. 2024). To more accurately evaluate ecological risks, it is essential to incorporate these additional exposure routes into risk assessment protocols.

## Supplementary Information

Below is the link to the electronic supplementary material.

ESM 1 (XLSX 45.0 KB)

ESM 2 (DOCX 16.3 KB)

## Data Availability

All raw data relevant to this study are provided in the supplementary material.
