# Supplementary material for: Effects of two nAChR agonists on wood ants: acetamiprid induces lethality and immediate hypoactivity, while flupyradifurone causes time-delayed hyperactivity
Source: Environ Sci Pollut Res Int. 2025 Jul 18;32(30):18366–79. doi: 10.1007/s11356-025-36755-z (PMC12328479; doi:10.1007/s11356-025-36755-z)
Supplement: Supplementary file 2 — (DOCX 16.3 KB) [file 11356_2025_36755_MOESM2_ESM.docx]

Exemplary code for statistical analysis of covered distances:

#check model fit

distance.null = lmer(distance ~ (1|subject)

+ (1|treatment)+(1|time), Distance_statistics_Acetamiprid,

REML = FALSE)

distance.model.interact = lmer(distance ~ treatment*time +

(1|subject) ,

data=Distance_statistics_Acetamiprid,

REML = FALSE)

distance.model = lmer(distance ~ treatment+time +

(1|subject),

data=Distance_statistics_Acetamiprid,

REML = FALSE)

anova(distance.null,distance.model)

anova(distance.null,distance.model.interact)

anova(distance.model,distance.model.interact)

AIC(distance.model)

AIC(distance.model.interact)

AIC(distance.null)

BIC(distance.model)

BIC(distance.model.interact)

BIC(distance.null)

# Residual plot

plot(residuals(distance.model.interact), col = "blue", ylab = "Residuals", xlab = "Observation Order")

abline(h = 0, col = "red", lty = 2)

# Predicted vs observed plot

plot(fitted(distance.model.interact), Distance_statistics_Acetamiprid$distance, col = "blue", xlab = "Predicted Values", ylab = "Observed Values")

abline(a = 0, b = 1, col = "red", lty = 2)

# Diagnostic plot homogenity

plot(distance.model.interact, which = 1)

plot(robust_model, which = 1)

# Check for homogenity

residuals <- resid(distance.model.interact)

grouping_variable <- Distance_statistics_Acetamiprid$treatment

# Perform Levene's test on residuals

leveneTest(residuals ~ grouping_variable)

#QQ-Plot

qqnorm(resid(distance.model.interact))

qqline(resid(distance.model.interact))

hist(resid(distance.model.interact), col = "lightblue", main = "Histogram of Scaled Residuals")

# Fit a robust linear mixed-effects model

robust_model = rlmer(distance ~ treatment*time +

(1|subject) ,

data=Distance_statistics_Acetamiprid)

summary(robust_model)

# Effect sizes

robust_model <- rlmer(distance ~ treatment * time + (1 | subject), data = Distance_statistics_Acetamiprid)

resid_sd <- sd(residuals(robust_model))

cohen_d <- fixef(robust_model) / resid_sd_

print(cohen_d)

Exemplary code for statistical analysis of abnormal behaviors:

# Create models

glmm_model <- glmer(abnormal_behavior ~ time * treatment + (1 | subject),

family = binomial(link = "logit"),

data = AbnormalBehavior_statistics_Acetamiprid)

glmm_null <- glmer(abnormal_behavior ~ 1 + (1 | subject),

family = binomial(link = "logit"),

data = AbnormalBehavior_statistics_Acetamiprid)

glmm_wo <- glmer(abnormal_behavior ~ time + treatment + (1 | subject),

family = binomial(link = "logit"),

data = AbnormalBehavior_statistics_Acetamiprid)

# Check fit

AIC(glmm_model)

AIC(glmm_null)

AIC(glmm_wo)

BIC(glmm_model)

BIC(glmm_null)

BIC(glmm_wo)

#check assumptions

library(DHARMa)

simulation_output <- simulateResiduals(glmm_wo)

plot(simulation_output)

Exemplary code survival:

library(openxlsx)

library(survival)

#Plot survival curves

survival_object = Surv(time = KaplanMeier_Acetamiprid_336h_CSV$time, event= KaplanMeier $response)

fit= survfit(survival_object ~ dose, data=KaplanMeier)

plot(fit, col = c("green","yellow","orange","red","black"), lty = 1:5, xlab = "Time (hours)", ylab = "Survival Probability", main = "Kaplan-Meier Survival Curve")

summary(fit)

# Do a pairwise log-rank-test

install.packages("survminer")

library(survminer)

library(survminer)

pairwise_result <- pairwise_survdiff(Surv(time, response) ~ dose,

data = KaplanMeier,

p.adjust.method = "bonferroni")

pairwise_result

#Calculate effect sizes

cox_model <- coxph(Surv(time, response) ~ dose, data = KaplanMeier)

summary(cox_model)
